# Mothers facing greater environmental adversity experience increased costs of reproduction

**DOI:** 10.1126/sciadv.adz6422

**Published:** 2025-11-07

**Authors:** Euan A. Young, Erik Postma, Virpi Lummaa, Hannah L. Dugdale

**Affiliations:** ^1^ Groningen Institute for Evolutionary Life Sciences, University of Groningen, Groningen, Netherlands.; ^2^ Centre for Ecology and Conservation, University of Exeter, Penryn, UK.; ^3^ Department of Biology, University of Turku, Turku, Finland.

## Abstract

Evolutionary theory of aging predicts that women with increased reproductive effort live shorter lives, but evidence is inconsistent. These inconsistencies could be because environmental conditions influence how much a mother’s life span is reduced when having more children, i.e., their life-span cost of reproduction. Using a structural equation measurement model, we compare how reproductive effort affects the life span of 4684 women exposed across different life stages, or not at all, to the Great Finnish Famine. We find that life-span costs of reproduction became higher in mothers exposed to the famine during reproduction and, for these mothers, amounted to lower life expectancies of ~0.5 years per child. Conversely, reproduction did not shape the life spans of mothers not exposed to the famine or exposed postreproduction or during development. This natural experiment reveals how environmental adversity can influence reproductive costs, providing a biological explanation for previous inconsistent findings while showing how reproductive behavior has shaped the evolution of aging in humans.

## Teaser

Mothers with more children during a famine had shorter lives, suggesting that harsh conditions increase costs of reproduction.

## INTRODUCTION

Understanding why we age—defined as a physiological decline in later life—and why some of us undergo this aging process faster than others are vital foundations for improving human health spans (*1*). One theory—the disposable soma hypothesis—predicts that aging is a consequence of natural selection honing our physiology to maximize reproduction at the cost of other organismal functions, resulting in an accumulation of damage through life that limits life spans (*2*, *3*). Consequently, researchers have tested whether individuals who devote more resources to reproduction have shorter life spans (*4*). However, expected negative associations between reproduction and life spans are rarely found (*5*, *6*) and typically only when using experimental manipulations [e.g., (*7*)]. For humans, the bearing and raising of even one child is energetically demanding for women (*8*), but—despite more than 100 years of research (*9*)—evidence of a reproduction-survival trade-off is inconsistent (*10*–*12*), and the role that human reproductive behavior plays in shaping aging remains unclear.

One potential explanation for these inconsistencies is that the reproduction-survival trade-off does not manifest itself in the same way when measured across a heterogeneous set of individuals (*13*). First, individual heterogeneity will weaken the observed slope of the regression of survival against reproduction (i.e., the estimate of the reproduction-survival trade-off function) because among-individual variation in resource acquisition introduces a positive association between reproductive effort and life span that can mask the within-individual reproduction-survival trade-off [i.e., the big house and big car syndrome (*14*, *15*)]. Thus, the estimated trade-off function quantifies how important reproductive behavior versus individual heterogeneity is in shaping human life spans, and studies aim to account for such individual heterogeneity in their analyses (*16*). Second, among-individual variation (e.g., in frailty or quality), due to intrinsic or environmental differences, may mean that some women will manifest costs of reproduction more than others (*12*, *13*, *17*–*19*). Previously, the reproduction-survival trade-off has been found to be stronger in lower socioeconomic status women (*20*, *21*) and women experiencing higher levels of infant mortality (*22*). However, we still have a poor understanding of which biological factors drive these changes in the reproduction-survival trade-off, with studies showing considerable variation in the estimated trade-off within the same populations over time, in line with environmental causes playing a role (*23*–*25*). Understanding these drivers can give us insight into the mechanisms underlying this fundamental trade-off, how it manifests itself, and under what circumstance human reproductive behavior is an important factor in shaping aging.

Although the role of reproductive effort in shaping aging is uncertain, examples of environmental effects on human health are numerous (*26*, *27*). In humans, experiencing periods of extreme environmental adversity, such as famines, can accelerate the biological aging process (*28*). Famine can have an array of effects, from long-term individual effects on human health, such as prenatal famine exposure increasing risk of type 2 diabetes and schizophrenia later in life (*29*, *30*), to social and economic consequences (*31*). Although widely used as natural experiments in other fields, the role that famine exposure plays in shaping estimated life-span costs of reproduction has not, to our knowledge, been studied.

Given the well-documented adverse effects of famines, mothers exposed to famines could show greater reductions in life span for a given increase in reproductive effort. However, this reduction may depend on the life history stage of the mother during the famine exposure. First, even short-term fluctuations in early-life environmental conditions can lead to physiological differences (*32*) that influence the overall life span and realized reproduction of individuals (*33*, *34*). Thus, women experiencing famines during development may show increased costs of reproduction. However, Nenko *et al.* (*24*) found no evidence that crop yields, spring temperatures, or infant mortality rates at birth modified the reproduction–life span trade-off in mothers. Instead, mothers may be especially vulnerable to resource limitations while raising children (*24*), due to both the calorific demands of pregnancy and lactation and increased vulnerability to disease (*8*). Wang *et al.* (*25*) proposed that some individuals experiencing the Great Depression and Second World War while raising and bearing offspring caused the variation in costs of reproduction in the Framingham cohort study. Last, women in postreproductive life are particularly at risk of mortality under lower food availability (*34*). Thus, mothers could suffer increased frailty due to high reproductive effort, but perhaps only when combined with famine exposure during postreproductive life would this result in reduced life spans. However, no study has examined how a period of environmental adversity affects life-span costs of reproduction in humans and whether this might vary across life stages.

Rigorous tests of these hypotheses require detailed individual-level life history data for a period encapsulating a major famine event. Here, we use individual life history data across 250 years from rural Finland to quantify life-span costs of reproduction and compare how they changed across mothers exposed to the Great Finnish Famine in different life stages and those not exposed at all. In the 1860s, Finland experienced several harsh winters resulting in a series of low harvests, which, in 1867, dropped to catastrophic levels, resulting in a major loss of employment (*35*). Widespread migration then followed as people went in search of work, which in turn triggered a large spike in infectious diseases, such as typhoid fever, typhus, and dysentery (*35*). This led to an excess mortality of 110,000 [or 5 to 10% of Finland’s population (*36*)] making it one of the most devastating famines in recent European history and the worst in Finland since the 17th century (*37*).

**Fig. 1.**
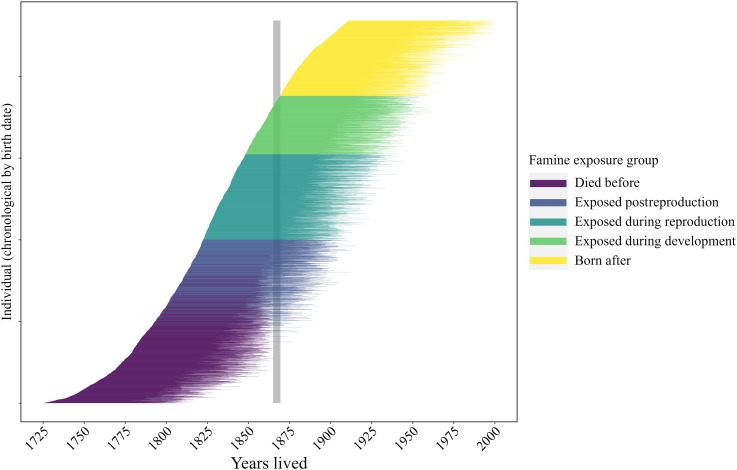
Line graph showing the years lived for each mother included in the analyses, sorted chronologically by their birth date (*N* = 4684). Colors indicate their exposure to the Great Finnish Famine (gray bar) and groupings in the analyses: died before (*n* = 1297), exposed postreproduction (*n* = 705), exposed during reproduction (*n* = 1044), exposed during development (*n* = 716), and born after (*n* = 922).

Here, we capitalize on the fact that this famine occurred in the middle of our study period, allowing us to estimate the life-span costs of reproduction before, during, and after a famine. Specifically, we compare mothers exposed to the famine during three life stages [during their development (ages 0 to 19), while reproductively active (ages 19 to 45), and while in postreproductive life (ages 45+)] to mothers dying before the famine and those born in the years after using a structural equation measurement model (*N* = 4868; Fig. 1). We follow Helle (*38*) and use a measurement model using multiple indicator variables to obtain a comprehensive measure of lifetime reproductive effort. Within this structural equation model (SEM) framework, we estimate the association between reproductive effort and life span (i.e., the reproduction-survival trade-off function) in each famine exposure group and how these life-span costs of reproduction differed across groups, accounting for the changes in reproductive effort and life span in relation to famine exposure. Broadly, we expected mothers exposed to the famine to show an increased life-span cost of reproduction but that this cost would depend on the life stage of mothers during this famine exposure. Showing such a dependency would shed light on why the estimated costs of reproduction are so variable both among and within studies.

## RESULTS

### Life-span costs of reproduction

**Fig. 2.**
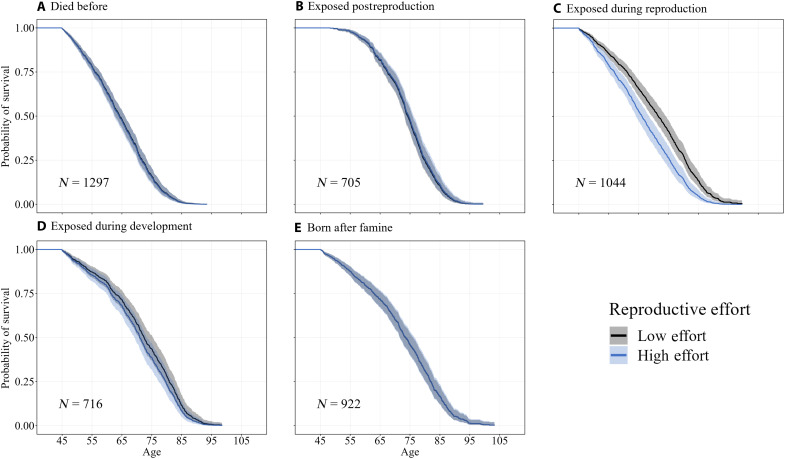
Predicted survival curves for low and high reproductive effort across five famine exposure groups. Curves were generated using Cox proportional hazards models on each famine exposure group separately (see table S1). Each panel represents a different famine exposure group: (**A**) died before famine, (**B**) exposed postreproduction, (**C**) exposed during reproduction, (**D**) exposed during development, and (**E**) born after famine. Survival curves are shown for low (fifth percentile, −3.8) and high (95th percentile, 6.5) reproductive effort levels. All curves are calculated for individuals of intermediate socioeconomic status and median relative birth date within each famine group. Shaded areas represent 95% CIs.

Exposure to the Great Finnish Famine shaped the estimated reproduction-survival trade-off (Fig. 2 and Tables 1 and 2). Specifically, increases in reproductive effort were associated with a reduction in life span in mothers exposed to the famine while reproductively active, but not among other groups (Fig. 2 and Table 2). Mothers exposed to the famine who had one child would be expected to live to age 71.6 [95% confidence intervals (CIs) = 69.2 to 74.6], whereas a mother with 15 children would only be expected to live to age 64.3 (61.7 to 68.3) or a reduction in life span of half a year per child (Fig. 2 and table S1). In line with this, pairwise comparisons of the five groups showed that the association between reproductive effort and life span in mothers exposed during reproduction was also higher than in mothers dying before the famine, mothers born after the famine, and mothers exposed to the famine in postreproductive life (*P* < 0.05) and marginally higher than those exposed to the famine during development (*P* = 0.087; Table 2 and Fig. 2).

**Table 1.** The effects on lifetime reproductive effort and life span (as measured by log-hazard mortality risk) from a structural equation model of 4684 mothers. Factor loadings, mean values (intercept), and residual variance for indicator variables used to estimate lifetime reproductive effort in the measurement model are shown. Estimates are shown with 95% CIs and *P* values; x represents an interaction. Significant effects (*P* < 0.05) are in bold. Marginally significant effects (*P* > 0.05 but < 0.10) are shown in italics. Effects of famine exposure are compared to women who died before the famine. Socioeconomic statuses are compared to intermediate statuses. Relative birth date is scaled by year and centered by the middle birth date for each group.

Variable	Estimate (95% CI)	*P* value
*Lifetime reproductive effort loadings*		
Number of children born	1 (1–1)	**–**
**Number of sons born**	**0.536 (0.523–0.548)**	**<0.001**
**Age at last reproduction**	**1.238 (1.187–1.289)**	**<0.001**
**Reproductive tenure**	**2.131 (2.092–2.17)**	**<0.001**
**Child-years experienced**	**3.878 (3.811–3.945)**	**<0.001**
**Number of multibirth children born**	**0.052 (0.044–0.059)**	**<0.001**
*Effects on reproductive effort*		
Exposure group (exposed postreproduction)	0.125 (−0.442–0.691)	0.666
Exposure group (exposed during reproduction)	*0.386 (−0.006*–*0.778*)	*0.054*
**Exposure group (exposed during development)**	**0.551 (0.080–1.022)**	**0.022**
**Exposure group (born after)**	**−0.504 (−0.922–0.086)**	**0.018**
Relative birth date	−0.004 (−0.013–0.004)	0.343
Relative birth date × exposure group (exposed postreproduction)	0.022 (−0.005–0.049)	0.108
**Relative birth date × exposure group (exposed during reproduction)**	**0.033 (0.008–0.057)**	**0.010**
Relative birth date × exposure group (exposed during development)	0.019 (−0.018–0.057)	0.308
**Relative birth date × exposure group (born after)**	**−0.061 (−0.078–0.044)**	**<0.001**
**Socioeconomic status (lower)**	**−0.541 (−1.020–0.062)**	**0.027**
Socioeconomic status (lower) × exposure group (exposed postreproduction)	−0.470 (−1.228–0.287)	0.224
**Socioeconomic status (lower) × exposure group (exposed during reproduction)**	**−0.653 (−1.299–0.008)**	**0.047**
Socioeconomic status (lower) × exposure group (exposed during development)	−0.132 (−0.875–0.611)	0.728
Socioeconomic status (lower) × exposure group (born after)	−0.335 (−1.010–0.339)	0.330
**Socioeconomic status (upper)**	**0.609 (0.243–0.975)**	**0.001**
Socioeconomic status (upper) × exposure group (exposed postreproduction)	−0.010 (−0.594–0.574)	0.974
Socioeconomic status (upper) × exposure group (exposed during reproduction)	−0.113 (−0.655–0.429)	0.683
Socioeconomic status (upper) × exposure group (exposed during development)	−0.265 (−0.890–0.359)	0.405
**Socioeconomic status (upper) × exposure group (born after)**	**−0.583 (−1.157–0.008)**	**0.047**
*Effects on mortality risk*		
**Exposure group (exposed postreproduction)**	**0.333 (0.283–0.392)**	**<0.001**
**Exposure group (exposed during reproduction)**	**0.694 (0.593–0.812)**	**<0.001**
**Exposure group (exposed during development)**	**0.600 (0.508–0.711)**	**<0.001**
**Exposure group (born after)**	**0.462 (0.397–0.538)**	**<0.001**
Lifetime reproductive effort	1.003 (0.985–1.021)	0.733
Lifetime reproductive effort × exposure group (exposed postreproduction)	0.994 (0.966–1.023)	0.682
**Lifetime reproductive effort × exposure group (exposed during reproduction)**	**1.041 (1.009–1.073)**	**0.011**
Lifetime reproductive effort × exposure group (exposed during development)	1.009 (0.977–1.041)	0.599
Lifetime reproductive effort × exposure group (born after)	0.996 (0.964–1.028)	0.794
**Relative birth date**	**1.026 (1.022–1.029)**	**<0.001**
Relative birth date × exposure group (exposed postreproduction)	1.005 (0.998–1.012)	0.161
**Relative birth date × exposure group (exposed during reproduction)**	**0.970 (0.962–0.980)**	**<0.001**
**Relative birth date × exposure group (exposed during development)**	**0.972 (0.961–0.984)**	**<0.001**
**Relative birth date × exposure group (born after)**	**0.960 (0.954–0.967)**	**<0.001**
**Socioeconomic status (lower)**	**1.305 (1.095–1.553)**	**0.003**
Socioeconomic status (lower) × exposure group (exposed postreproduction)	1.027 (0.811–1.303)	0.822
Socioeconomic status (lower) × exposure group (exposed during reproduction)	1.130 (0.878–1.455)	0.342
**Socioeconomic status (lower) × exposure group (exposed during development)**	**0.752 (0.579–0.976)**	**0.032**
Socioeconomic status (lower) × exposure group (born after)	0.866 (0.674–1.113)	0.260
Socioeconomic status (upper)	1.110 (0.974–1.264)	0.118
Socioeconomic status (upper) × exposure group (exposed postreproduction)	0.967 (0.801–1.165)	0.720
Socioeconomic status (upper) × exposure group (exposed during reproduction)	0.873 (0.715–1.066)	0.183
Socioeconomic status (upper) × exposure group (exposed during development)	0.872 (0.705–1.078)	0.206
Socioeconomic status (upper) × exposure group (born after)	1.121 (0.91–1.381)	0.282
*Intercepts*		
**Number of children born**	**5.265 (4.978–5.552)**	**<0.001**
**Number of sons born**	**2.695 (2.539–2.852)**	**<0.001**
**Age at last reproduction**	**38.133 (37.762–38.504)**	**<0.001**
**Reproductive tenure**	**11.872 (11.254–12.49)**	**<0.001**
**Child-years experienced**	**21.131 (20.016–22.245)**	**<0.001**
**Number of multibirth children born**	**0.211 (0.187–0.235)**	**<0.001**
*Residual variances*		
**Number of children born**	**0.344 (0.276–0.411)**	**<0.001**
**Number of sons born**	**1.348 (1.283–1.412)**	**<0.001**
**Age-at-last reproduction**	**19.315 (18.463–20.166)**	**<0.001**
**Reproductive tenure**	**10.771 (10.128–11.415)**	**<0.001**
**Child-years experienced**	**27.365 (24.856–29.875)**	**<0.001**
**Number of multibirth children born**	**0.439 (0.397–0.481)**	**<0.001**
**Lifetime reproductive effort**	**8.298 (7.995–8.602)**	**<0.001**

**Table 2.** Pairwise comparisons of the association between reproductive effort and mortality risk across famine exposure groups. Diagonal values show the hazard ratio (95% CIs) for the effect of reproductive effort on mortality within each group. Off-diagonal values show *P* values for pairwise differences in this association between groups. Significant differences or associations (*P* < 0.05) are highlighted in bold; marginally significant associations (*P* > 0.05 and *P* < 0.10) are italicized. Results are from a structural equation model of the same structure as Table 1 but with different reference groups to estimate *P* values and associations.

	Died before famine	Exposed postreproduction	Exposed during reproduction	Exposed during development	Born after famine
**Died before famine**	1.003 (0.985–1.021)				
**Exposed postreproduction**	0.682	0.997 (0.975–1.019)			
**Exposed during reproduction**	**0.011**	**0.007**	**1.044 (1.018–1.069)**		
**Exposed during development**	0.599	0.404	*0.087*	1.012 (0.986–1.039)	
**Born after famine**	0.794	0.926	**0.018**	0.5	0.999 (0.972–1.026)

These results were confirmed in a complementary mixed model analysis that accounted for significant variation in life span among birth years, families, and 13 geographical regions (table S2). Mothers again varied in the reproduction-survival trade-off function according to famine exposure, with the statistically significant association between reproductive effort and life span only found in mothers exposed to the famine during reproduction and similar in magnitude as estimated in the previous model (tables S3 and S4). The association between reproductive effort and life span in mothers exposed to the famine while reproductively active was also higher than in all other groups, except for those exposed during development (table S4).

### Temporal changes in life span

There were differences among famine exposure groups in mean life span and in how life span changed over time within each group (Table 1 and table S1). Life expectancy for mothers dying before the famine was 66 years for those born in 1777 but declined over time to 55 years for mothers born in 1819 (Table 1 and table S1). Similarly, for mothers exposed to the famine postreproduction, life expectancy was 79 years for those born in 1796 but declined overtime to 70 years for mothers born in 1822 (Table 1 and table S1). These trends reflect the classification of these groups: Within mothers who died before the start of the famine, those born later must have lived shorter lives. Similarly, by focusing only on famine survivors, mothers born earlier among those exposed to the famine during postreproduction cannot have had short lives, with the higher life expectancies overall likely reflecting the loss of higher frailty women during the famine itself. Mothers exposed to the famine during reproduction had a life expectancy of 71 years, which stayed similar for mothers exposed during development and did not change through time in either group (Table 1 and table S1). However, life expectancies then increased for mothers born after the famine from 71 years in mothers born in 1869 to 78 years for mothers born in 1910 (Table 1 and table S1).

### Temporal changes in reproductive effort

Mean reproductive effort did not differ between mothers dying before the famine and those exposed to the famine postreproduction, and it remained constant overtime within each group (Table 1 and fig. S1). However, for mothers exposed during reproduction, reproductive effort increased from being similar to prefamine levels for mothers born in 1822 to being 0.8 children higher in mothers born in 1848 in this group (Table 1). Reproductive effort then remained similarly high for mothers exposed during development who had on average 0.6 children more than prefamine levels, which did not change over time (Table 1). Reproductive effort then declined rapidly in mothers born after the famine going from mothers born in 1869 having 0.8 children more than mothers dying before the famine to mothers born in 1911 having 1.8 children fewer than prefamine levels (Table 1).

### Socioeconomic status effects

Socioeconomic status did not modify the association between reproductive effort and life span (table S5). However, there were differences in both life span and reproductive effort across socioeconomic status groups, with the magnitude of these differences changing according to famine exposure (Table 1 and table S1). No differences in life spans were found in mothers dying before the famine between higher and middle socioeconomic statuses, and these effects did not change across famine exposure groups (Table 1). However, lower socioeconomic status mothers had lower life expectancies of around three years compared with intermediate socioeconomic status mothers (Table 1). This difference in life expectancies between lower and intermediate socioeconomic status mothers remained similar across famine exposure groups, except in lower socioeconomic status mothers exposed to the famine during development who did not have lower life spans than mothers of intermediate socioeconomic status (Table 1).

Upper socioeconomic status mothers dying before the famine had higher reproductive effort than mothers of intermediate socioeconomic status, amounting to 0.6 more children (Table 1). These differences gradually decreased through time until mothers born after the famine, where upper and intermediate socioeconomic status mothers had identical reproductive effort (Table 1). Mothers from lower socioeconomic statuses had lower reproductive effort amounting to 0.6 fewer children in mothers dying before the famine than those from intermediate socioeconomic status. This difference in reproductive effort between lower and intermediate socioeconomic status mothers remained stable, except for mothers exposed during reproduction, where the difference increased to 1.2 fewer children.

## DISCUSSION

Using life history records for 4684 Finnish women, we find evidence supporting adverse environmental conditions increasing the importance of reproductive behavior in shaping a mother’s life span (Fig. 2 and Table 2). Specifically, mothers exposed to the Great Finnish Famine (1866–68) while reproductively active showed reduced life spans of ~0.5 years per child as estimated through a structural equation measurement model. On the other hand, mothers not exposed to the famine (i.e., mothers who died before or were born after) or those exposed to the famine in postreproductive life or during development did not pay a life-span cost of reproduction. Although the effects of famines on human health and aging are well documented (*28*), with some evidence for environmentally dependent costs of reproduction in animals (*13*, *19*), we provide evidence of a famine event modifying the reproduction-survival trade-off function in humans. These findings therefore add a biological explanation—to previously highlighted methodological explanations [detailed in (*39*) and (*40*)]—for why several studies have shown variation in costs of reproduction across cohorts (*23*–*25*): Fluctuations in environmental conditions experienced by mothers while reproductively active affect the trade-off function. This strengthens the evidence for the existence of life-span costs of reproduction and gives insight into under what circumstances human reproductive behavior is an important driver of aging.

These results align with other studies showing variation in the estimated reproduction-survival trade-off among mothers in human populations. For instance, one recent study using data for preindustrial Canada showed that only mothers having both high reproductive effort (i.e., a high number of sons) and high lifetime infant mortality showed increased life-span costs of reproduction (*22*). While this change in the reproduction-survival function across mothers is in line with our findings, Invernizzi *et al.* (*22*) did not explore how environmental conditions drive variation in infant mortality among mothers beyond showing an effect of a mother’s birth year [see table S1 in (*22*)]. These results also show parallels with studies showing that women of lower socioeconomic status showed a stronger trade-off (*20*, *21*). However, while these results align with some previous findings, we present the first evidence suggesting that environmental adversity influences the reproduction-survival trade-off across women.

Our finding that only mothers exposed to the famine while reproductively active showed life-span costs of reproduction could be due to the increased energetic demands of bearing and raising children during a period of high environmental adversity, as previously suggested (*41*). When pregnant, mothers must increase their caloric consumption by, on average, 300 kcal/day to sustain a healthy pregnancy, which rises to 460 to 630 kcal/day during breastfeeding (*42*). If the mother’s pregnancy or breastfeeding coincides with a famine, it could mean that these increases in metabolic demands are paid by themselves through lowering basal metabolism and thus slowing or shutting down other important functions, such as immunity (*11*, *43*), resulting in a decline in health and shorter life spans. Specifically, circulating estrogen levels—which are important for cardiovascular health (*44*)—are very low during breastfeeding (*45*). These levels are even lower in mothers with poor nutrition (*46*), which could mean that cardiovascular disease risk [which is already a risk factor of high parity (*47*)] could be even higher in mothers of high reproductive effort experiencing poor nutrition while reproducing. Although we can speculate, ultimately, the underlying mechanisms are difficult to disentangle in genealogical data, but we hope that these results may help guide future human biobank studies or experiments on model species where unraveling underlying mechanisms is more attainable.

In addition to famine exposure, we found that demographic and cultural factors were important drivers of reproductive effort, life span, and their association. Differences across socioeconomic groups were pronounced, with lower socioeconomic status mothers having lower realized reproduction (*37*) and shorter lives (*48*) but not increased life-span costs of reproduction [table S5 and (*38*) but see (*21*)]. We also found that mothers exposed to the famine during reproduction lived shorter lives than those exposed during development, and those exposed during development lived shorter lives than mothers born after the famine. Rather than being a direct effect of famine exposure, this is probably a symptom of increased industrialization, which, in line with country-wide trends, started during this period (*49*). These improvements in life span through, for example, vaccination (*50*), combined with a reduction in overall reproductive effort, are potential additional reasons why the importance of the reproductive-survival trade-off decreased in mothers born after the famine. This is analogous to results from the Netherlands, where the reductions in life span for mothers having more children found in 1850 had disappeared by 1910 (*51*). However, these results require replication outwith northern European countries.

Although high reproductive effort during the famine may have affected individual health directly, among-individual processes, such as selective disappearance, may still have influenced the reproduction-survival trade-off function. Because the association between reproduction and survival will weaken with higher individual heterogeneity (*14*, *15*, *17*), higher mortality rates during the Great Finnish Famine may have driven higher mortality of frailer individuals [as suggested by a previous study (*52*)], reducing the overall amount of individual heterogeneity in mothers sampled after completing reproduction and increasing the strength of the association. However, because our study necessarily included only survivors of the famine, selective disappearance due to the famine could have occurred in all of the famine exposure groups. Moreover, given that the effects of the famine on mortality were biased toward the very young and old and lowest among reproductive age individuals (*53*), the selective disappearance should have been highest in the development and postreproductive famine exposure groups—even after accounting for adverse conditions potentially increasing maternal death during childbirth (*54*). However, we found that the trade-off function was strongest and significant only in mothers reproducing during the famine, further suggesting that our results reflect a direct negative within-individual effect of famine exposure on reproducing mothers. Thus, while it is possible that among individual factors, such as selective disappearance, may have shaped some of our findings, the evidence suggests that it is not the primary cause of the stronger reproduction-survival trade-off in mothers exposed to the famine during reproduction.

However, higher selective disappearance in the mothers exposed during development may help explain why no moderating effects of the early-life environment have been found here and elsewhere (*24*). For instance, if selective disappearance removes the frailest mothers who are most susceptible to the trade-off, leaving a more robust subset of mothers for whom reproduction is less costly, then the trade-off function may become weaker with greater selective disappearance. Some evidence of increased selective disappearance in the mothers exposed during development may be visible in our results, with women of lower socioeconomic status in general having lower life span, except if they have been exposed to the famine during development where selective disappearance may be larger. While evidence for cohorts in gestation during the Great Finnish Famine having shorter lives is lacking (*55*), given the importance of the early-life environment on human health (*56*), and in modifying the reproduction-survival trade-off function in wild animal populations (*57*), future studies should aim to better account for selective disappearance when studying how environmental adversity may modify life-span costs of reproduction, particularly when looking for early-life effects.

We emphasize that our findings should be interpreted within the context of this being an observational study occurring over a single famine event that will have coincided with other historical events that may have shaped reproduction and life span. Albeit the largest [as measured by death rates (*37*)], the Great Finnish Famine is only one of many societal perturbations faced by Finns between the 18th century and present day. Principally, the Finnish War (1808–1809), severe epidemics and crop failures (1832–1833), the Finnish Civil War (1918), and World War II may all have shaped the reproduction and longevity of individuals in our study population (*58*). Although we were able to account for some demographic trends in reproductive effort and life span both within and among famine groups, including expanding analyses to account for correlations among birth years, regions, and families, which did not affect results, there are limitations. Because Finland began to industrialize soon after the famine (i.e., in the 1870s), we had no group of women born after the famine whose demography was comparable to the time preceding the start of industrialization. Thus, proving the causality of the Great Finnish Famine (1866–68) shaping the trade-off function is difficult within our system, but it may be possible in other datasets.

After more than 100 years of research, human observational studies have provided mixed evidence for life-span costs of reproduction, leading some to believe that reproductive behavior is not important in shaping human aging. Contrary to this, we provide evidence for a life history trade-off between reproductive effort and life span and demonstrate that—under harsh conditions—reproductive effort is an important driver of individual variation in life span. These findings suggest that famines can provide fitness costs to mothers in the form of reduced life span but may also include reduced reproduction of the children born from pregnancies during famine, although whether early-life environmental effects cause positive or negative effects fitness effects in humans remains debated (*59*, *60*). Although reproductive behavior may shape the life spans of mothers exposed to adverse environments while reproducing, it appears less important in contemporary Finland where investment in reproduction is far lower, and modern medicine may alleviate many potential costs. Last, we would like to acknowledge that because of unaccounted variation in individual heterogeneity, our study will still underestimate the underlying trade-off, and future research should focus on the underlying mechanisms or within-individual measures of aging [e.g., (*61*)]. Life span is the ultimate outcome of many underlying factors, and reproductive behavior in particular can have different effects on different diseases: For example, high reproductive effort increases the risk of cardiovascular disease (*47*), but very low reproductive effort may increase the risk of breast cancer (*62*). Therefore, future studies focusing on specific diseases or using biomarkers would be best placed to study how individual reproductive behavior shapes aging.

## MATERIALS AND METHODS

### Study population

To investigate how a mother’s reproductive behavior affects her life span, we used long-term individual life history data from rural Finland. Data were adapted from Parish records across rural Finland dating back to the 16th century. These records were maintained by the Lutheran Church and include not only birth, marriage, and death certificates but also yearly attendances of religious services, which, from 1749 onward, were required by law to be recorded for tax purposes. This allows complete life histories to be precisely constructed, including the migration of individuals between parishes. These individual records were then linked across generations resulting in a genealogical dataset of 100,598 people born from 1536 to 2012.

Most individuals in these data were born in a preindustrial period where agriculture employed 90% of the population (*35*). Preindustrial farming techniques, combined with particularly tough crop growing conditions in Finland, meant that during this period, yearly yields of the two main crops, rye and barley, varied a lot with the climatic conditions (*33*). Consequently, the population was characterized by high birth and death rates due to limited access to health care and contraception and infectious diseases such as smallpox, typhus, and typhoid fever (*58*). In our records, mothers on average had four children, but 38% of children died before their 15th birthday. On average, women had their first child at age 25, and 95% had their last child before age 45. Like other preindustrial European populations, the mating system was patrilocal and highly monogamous. Divorce was forbidden (*63*), and infidelity severely punished (*64*), with recent genetic studies showing that ~1% of children were born outside of marital ties (*65*, *66*).

Industrialization began in Finland in the 1870s—relatively late by European standards—and developed gradually, with manufacturing still employing only 20% of the population by 1910 (*35*). Throughout this period, access to health care improved, and vaccination of children against smallpox was relatively common by the 1880s (*50*). Consequently, life expectancy in Finland increased from 32 years at birth in 1865 to 1870 for those born in 1965 and is now above the European average (*67*). In this period, access to contraception also improved, and cultural preferences changed, with the fertility transition beginning around 1910 where birth rates declined from ~30 births per 1000 people to ~12 by the 1970s (*49*).

### Quantification of life spans

All data handling was performed in R 4.3.2 (*68*) within RStudio 2023.12.1 (*69*), using tidyr 1.3.1 (*70*). We calculated the life span of all individuals with a recorded birth (99%) and death (43%). For those with recorded years but missing months and/or days, the middle value was used (1 and 2% of recorded births and deaths, respectively). Individuals with missing death dates were then assigned a last-seen date of the final year they were known to have been alive in the data (available for 97% of individuals missing death dates), which could include a last-known birth of child or attendance of religious services. As the precise date of these services was often unknown, we standardized the date across individuals and treated all individuals as at least being alive on 1st January of their year of last appearance. Life spans were then calculated in decimal years using the lubridate package 1.9.2 (*71*) giving a median of 24 (range = 0 to 108). We also recorded whether this life span was calculated from known death dates or dates of last appearance which would be accounted for using a survival analysis (see the “Statistical analysis” section).

### Quantification of reproductive traits

We recorded six aspects of a woman’s reproductive history thought to capture a mother’s reproductive effort, defined as the total amount of resources devoted to bearing and raising offspring to independence (*38*). These variables included the four variables that Helle (*38*) used to measure reproductive effort: number of children born, number of child-years experienced, ages at last reproduction, and reproductive tenure (i.e., the time between first and last reproduction). In addition, we included the number of sons born and the number of children born that were twins or triplets (hence multiple births), which may be associated with increased reproductive effort (*72*). We also modified the definition of child-years experienced from the cumulative time that a mother cosurvived with her children while they were younger than age 18 to age 5, because the effort of bearing and raising offspring is relatively low and children are more independent after age 5 (*73*), even helping to raise younger siblings thereafter (*74*). Thus, if a mother survived at least 5 years after her last birth and had three children, one of which died at age 1, another age 10, and another age 60, then her child-years experienced would be (1 + 5 + 5 =) 11.

The median number of children for known mothers in our population was 3, ranging from 1 to 18. We estimated the ages at last reproduction and reproductive tenure for all mothers with a known child-birth date, which had medians of 35 (range, 16 to 51) and 8 (0 to 31), respectively. Sex of children was known in 99.4% of children, with mothers having 0 to 12 sons, with population-level birth sex ratios approximately 50:50. Multiple birth status was assigned to 99.3% of cases. Six percent of mothers had at least one set of twins or triplets, but one mother had three sets of twins. In cases where children or mothers had unknown death dates before age 5 (81%), the year of last appearance was used to calculate the child-years experienced. Median child-years experienced were 12 (0 to 75).

### Assigning famine exposure groups

All individuals were grouped according to whether—and in what life stage—they were exposed to the Great Finnish Famine. We first assigned the start and end dates of the famine as 1st February 1865 and 31st May 1869 which is the period of elevated excess mortality observed country-wide in Finland due to the famine (*35*). On the basis of these dates, we categorized all women surviving to adulthood (19.1 years, fifth percentile for age at first reproduction) into three groups: individuals dying before the famine (*n* = 3694), individuals exposed to the famine at some point in their live (*n* = 9337), and individuals born after the famine (*n* = 15,036). We then further divided individuals exposed to the famine into three groups based on when they were exposed to the famine: when they were developing, reproductively active, or postreproductive. We defined women as reproductively active during the famine if—at the midpoint of the famine (i.e., 31st March 1867)—they were older than the 5th percentile of age at first reproduction (19.1 years) but younger than the 95th percentile of age at last reproduction (44.9 years) calculated from mothers reproducing before the fertility transition in 1911 (*49*). Individuals younger than 19.1 years at the famine’s midpoint were assigned the developing group (*n* = 4231), and individuals older than 44.9 at the midpoint were assigned the postreproductive group (*n* = 1612), with those aged in between assigned to the reproductively active group (*n* = 3494). A total of 1052 women could not be assigned a famine category, due to missing birth or last seen dates.

### Assigning socioeconomic status groups

To account for a source of individual heterogeneity that can influence the life span and reproductive effort of women (*48*), we grouped women surviving to adulthood into three socioeconomic status groups: upper, including mostly landowners and merchants (*n* = 6591); intermediate, including mostly craftsmen, such as smiths, and fishermen (*n* = 7764); and lower, consisting of landless families and servants (*n* = 6750). In cases where a woman’s occupation was missing but the husband’s occupation was present, we assigned it based on the husband’s occupation (*n* = 2357). If both were present but different (*n* = 590), then the highest socioeconomic status was used. In total, socioeconomic status could be determined for 76% of adult women.

### Data selection

The data selection is summarized in fig. S2. For women surviving to adulthood, we first excluded childless women (*n* = 9275) because of the substantial differences in health of childless females in preindustrial populations that make comparisons to reproductive females (henceforth mothers) challenging (*12*). We then excluded mothers that had not been tracked throughout their entire reproductive period (*n* = 6658) to ensure the precise estimation of reproductive effort. We further excluded mothers who died before completing their reproductively active period (age 44.9, as defined above) because we wanted to estimate the long-term life-span costs of reproduction and minimize frailty heterogeneity (*n* = 2204). However, we accept that this may lead to an underestimation of the total costs of reproduction by excluding those who died directly after reproducing and by including only the most robust mothers that survived to postreproductive life (*23*, *40*). This left 9930 women. Before 1725, the coverage of the data was very low, and after 1911, there were increasing numbers of missing individuals because they are still alive, both of which could bias life spans, so were removed (*n* = 1906). We were also only interested in the long-term effects of the famine and so excluded those dying during the famine period itself (*n* = 478). We further controlled for individual heterogeneity by excluding mothers who were twins or triplets (*n* = 119), multiple marriers (*n* = 612), and women without recorded childbirth dates (*n* = 4) or socioeconomic statuses (*n* = 410) as done in other studies (*75*).

Last, before analyses, we examined the proportion of individuals with known death dates versus those that had only a last seen date (i.e., were censored) across the years lived for each of our famine exposure groups. Survival analyses (as used here) allow the combining of censored and uncensored data to estimate life spans but can become biased if the proportion of censoring is biased with respect to life span across predictors in a survival model (*76*). We found that the percentage of censored individuals was much higher in the groups of individuals exposed to the famine while reproductively active (43%), while developing (51%), and those born after the famine (35%), compared with those exposed postreproduction (4%) and dying before the famine (3%; fig. S3A), due to two spikes in the number of censored individuals between the years of 1898–1904 and 1910–11 (fig. S3B). Because of the temporal nature of our famine exposure groups, these resulted in systematic structuring of censoring across life spans (fig. S4), which would result in biased life spans. Removing life spans censored during those two periods (*n* = 1717) removed these biases (fig. S4) and resulted in a relatively even percentage of censored to noncensored individuals across famines exposure categories for analyses (2 to 11%; fig. S5).

This resulted in 4684 mothers with a median life span of at least 68 years (range, 45 to 103) and five children (1 to 17) (table S6). These values are slightly higher than the full dataset (see table S6) but remained comparable to other similar populations (*22*, *75*).

### Statistical analysis

Following Helle (*38*), we used a confirmatory factor analysis measurement model to estimate the latent lifetime reproductive effort from the six indicator variables that are hypothesized to be components of a woman’s reproductive effort (*77*–*79*). Measurement models use simultaneous equations to separate the correlation between these indicator variables into the true variance in reproductive effort and the error variance which captures the measurement error for each specific indicator variable (*80*). As indicator variables, we used the number of children born, number of child-years experienced, ages at last reproduction, and reproductive tenure, number of sons born, and number of children that were part of multiple births. Indicators with factor loading *P* < 0.05 contributed to estimating the lifetime reproductive effort. The number of children born was used to scale the latent reproductive effort so that each unit increase in reproductive effort could be interpreted as equivalent to the total resources used in raising one child. The latent reproductive effort was mean centered at 0 so values could be negative and indicate lower than average reproductive effort.

All reproductive variables significantly contributed to estimating reproductive effort (*P* < 0.001, loadings; Table 1). Specifically, increases in one unit of reproductive effort was associated with 0.536 (95% CIs = 0.523 to 0.548) more sons, 1.238 (1.187 to 1.289) years increase in ages at last reproduction, 2.131 (2.092 to 2.170) years longer reproductive tenures, 3.878 (3.811 to 3.945) years increase in child-years experienced, and 0.052 (0.044 to 0.059) more multiple births (Table 1). The number of children was clearly the most important component of the reproductive effort variable and showed a correlation with the estimated reproductive effort of 0.99 (see fig. S6).

This measurement model was incorporated within an SEM estimated using simultaneous equations and restricted maximum likelihood in the software Mplus 8.8 (*81*). This model was used to examine how reproductive effort, life span, and the direct effect of reproductive effort on life span changed across mothers exposed to the Great Finnish Famine. Life span was estimated using a semiparametric continuous-time survival model which approximates a Cox-proportional hazards model (*82*). This combines the number of years individuals were known to have lived and whether these individuals had a recorded death date or had been censored. By using a survival analysis, the SEM estimates how the log-hazard mortality risk changes according to each covariate. Hence, positive coefficients indicate an increased risk of mortality with an increase in the covariate and thus a reduction in survival probability or life span. The model included the direct effects of reproductive effort on life span while simultaneously estimating the differences in reproductive effort and life span across predictors. Separating these effects is a key strength of the SEM, which cannot be done in other models (such as linear mixed models). We also modeled the differences in life span and reproductive effort across socioeconomic statuses and temporal changes occurring in each famine exposure group by including a birth year effect relative to the middle birth year in each famine exposure group and by allowing the effects of relative birth year to vary across groups. We also tested whether the effect of reproductive effort on life span depended on socioeconomic status. *P* values less than 0.05 were deemed significant, and nonsignificant interactions were removed from the final models, least significant first, to aid interpretation of lower-order effects. Pairwise comparisons between categories were done by swapping the reference groups in the SEM. Collinearity of between variables used in models was checked using corvif () from Highstat 10 (*83*). The variance inflation factors were all <1.5. For illustration and quantification purposes, we extracted the estimated reproductive effort for mothers and ran Cox proportional hazard models with the same predictors for each famine exposure group separately, allowing us to estimate and present the predicted effect of changing reproductive effort on life span under a given famine exposure.

Mplus does not allow for the inclusion of random effects if some groups only have one sample (*81*). Therefore, we also used the extracted reproductive effort estimates for each mother from the SEM in mixed effect Cox proportional hazard models using Coxme 2.2-20 (*84*) to see if our results were robust when accounting for shared family, region, and birth year effects among mothers. For these analyses, we retained all but four individuals of unknown region. Mothers with unknown parents were assumed to have come from different families (44%) as sisters would have been identifiable in the data. Coxme models cannot model the direct effects of factors such as famine exposure on reproductive effort, but they represent a useful robustness check and still account for such dependencies when estimating the marginal effects. For Coxme models, we used likelihood ratio chi-square tests comparing models with and without a given predictor to test for the significance of predictors with a threshold of *P* < 0.05.

The SEMs were run in Mplus (*81*), implemented in R 4.3.0 (*68*) using MplusAutomation 1.1.0 (*85*). ggplot2 3.3.5 (*86*) and ggpubr 0.4.0 (*87*) were used for plotting, and ggeffects 1.5.1 (*88*) was used for estimating marginal effects. Survival 3.5.7 (*89*) was used for estimating life expectancies which were calculated as the predicted age at which survival probability was 50%, using median values for variables and an intermediate socioeconomic status if not otherwise specified.
