# Supplementary material for: Mothers facing greater environmental adversity experience increased costs of reproduction
Source: Sci Adv. 2025 Nov 7;11(45):eadz6422. doi: 10.1126/sciadv.adz6422 (PMC12594185; doi:10.1126/sciadv.adz6422)
Supplement: Supplementary file 1 — Figs. S1 to S6 Tables S1 to S6 [file sciadv.adz6422_sm.pdf]

Science Advances
AAAS

Supplementary Materials for

Mothers facing greater environmental adversity experience increased costs of reproduction

Euan A. Young *et al.*

Corresponding author: Hannah L. Dugdale, h.l.dugdale@rug.nl; Euan A. Young, e.a.young@rug.nl

Sci. Adv. **11**, eadz6422 (2025)
DOI: 10.1126/sciadv.adz6422

This PDF file includes:

Figs. S1 to S6
Tables S1 to S6

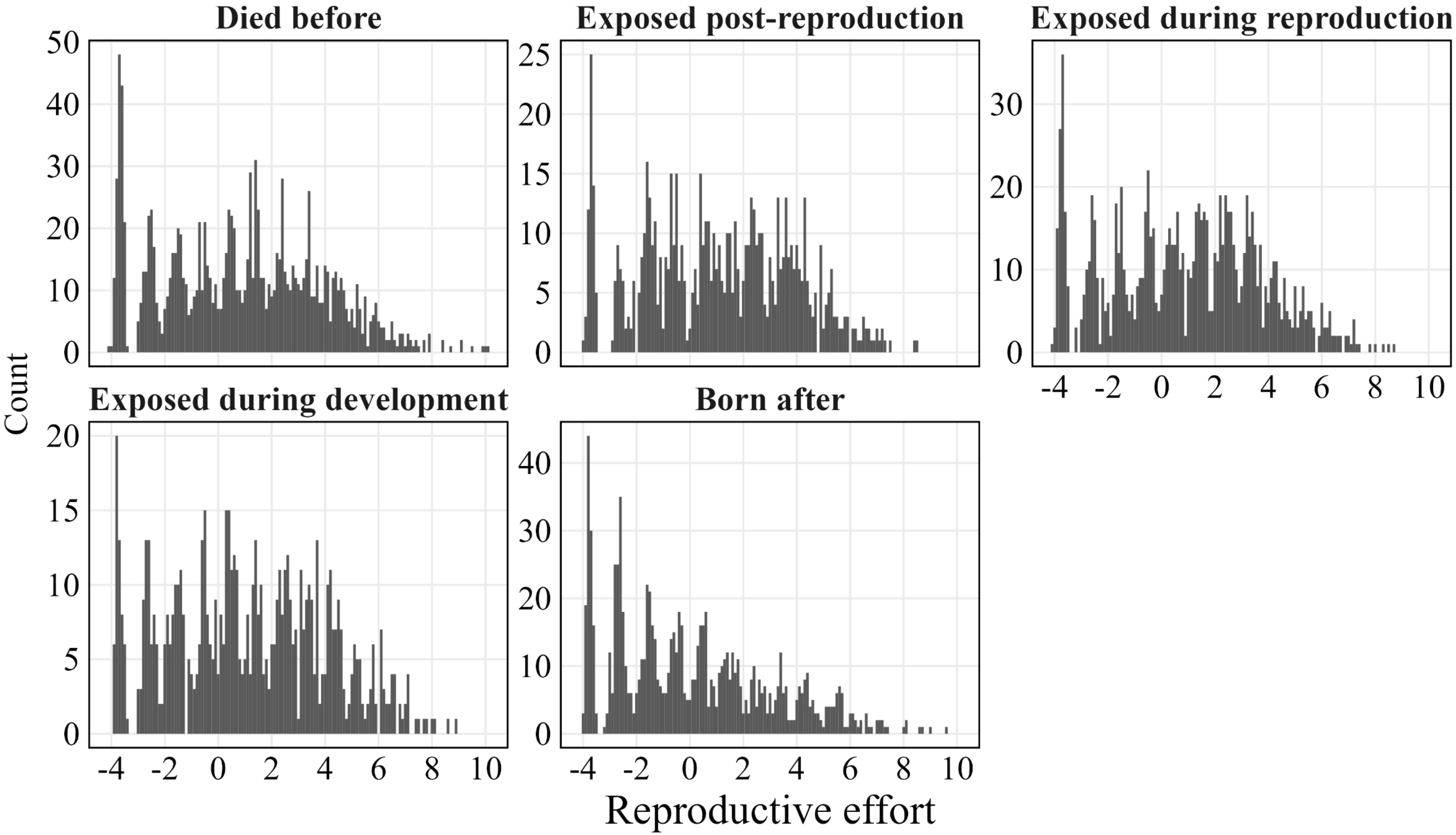

Figure S1: Histograms showing the distribution of the estimated reproductive effort variables in each famine exposure group.

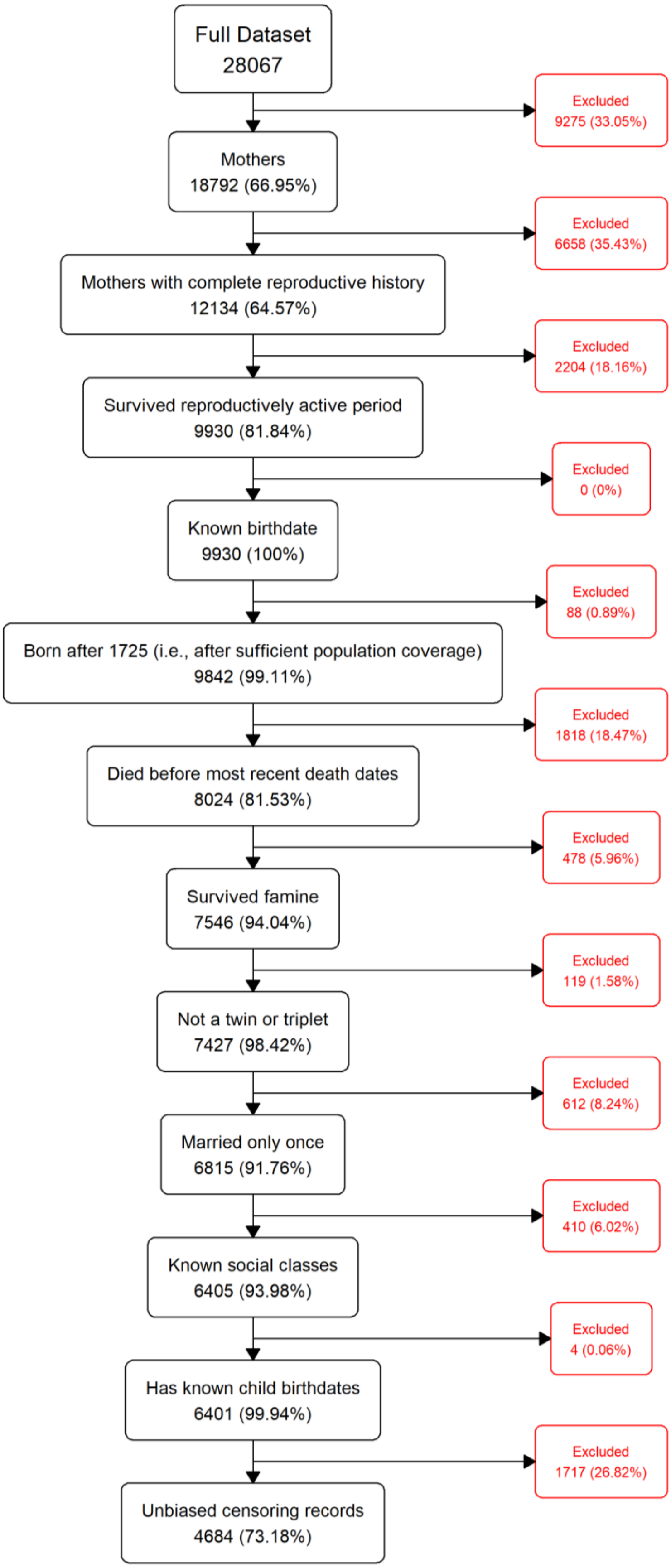

Figure S2: Data selection performed prior to analyses.

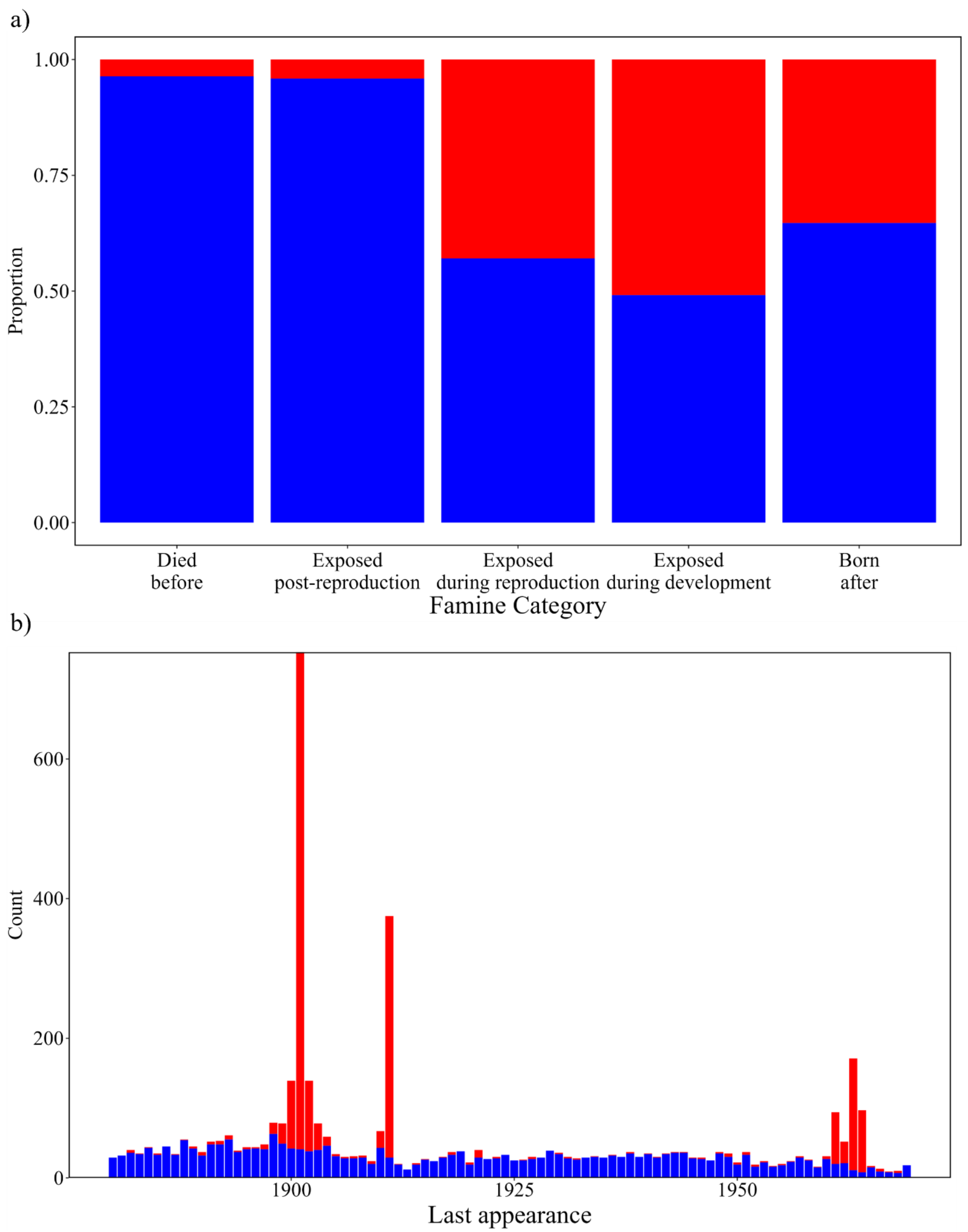

Figure S3: Bar charts showing (a) the proportion of censored (red) versus those with death dates (blue) in each group and (b) the number individuals censored across years of last appearance from 1880-1970 prior to removing censoring biases.

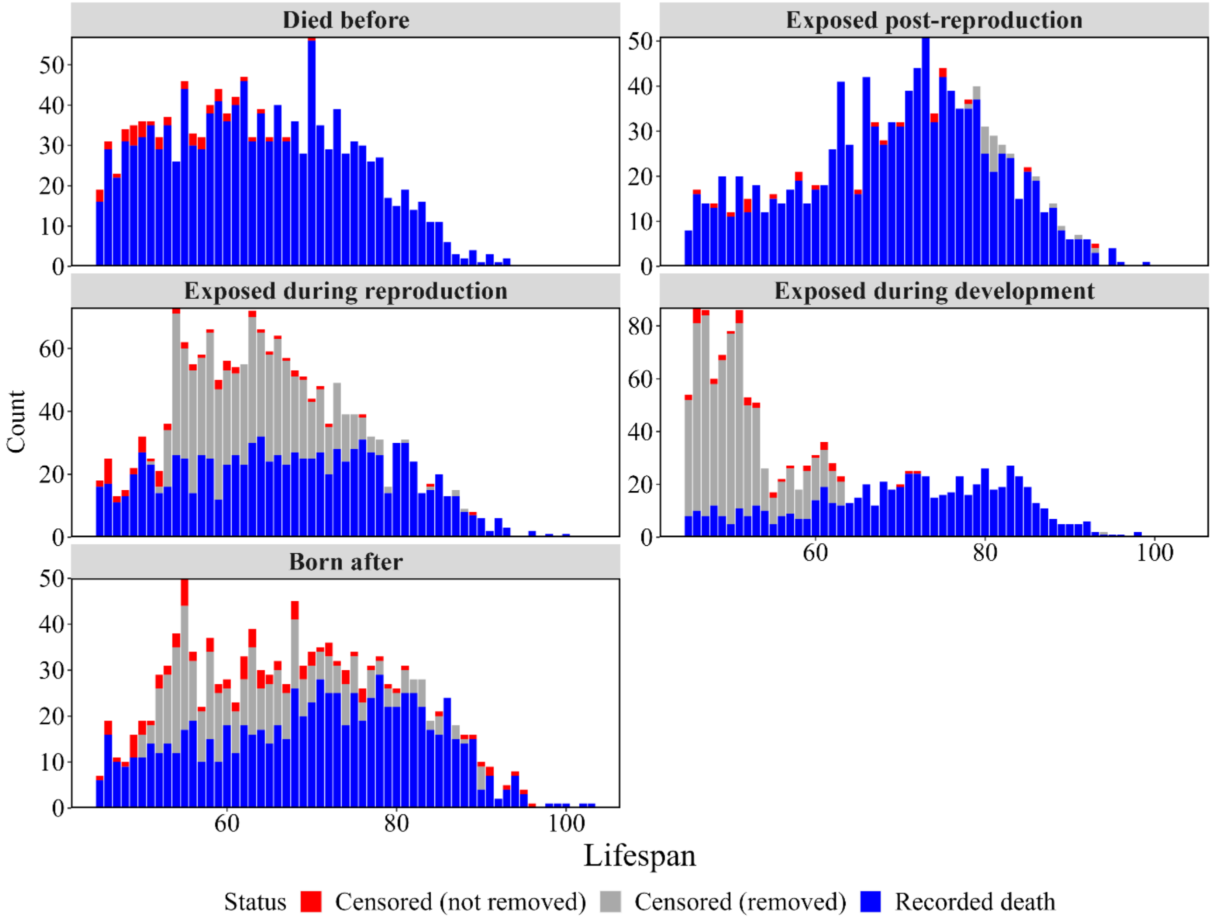

Figure S4: Bar charts showing the number of censored individuals that were not removed from analyses (red) vs those which were removed (grey) and those with recorded death dates (blue) across ages at date of last seen. Y-axes limits vary across groups.

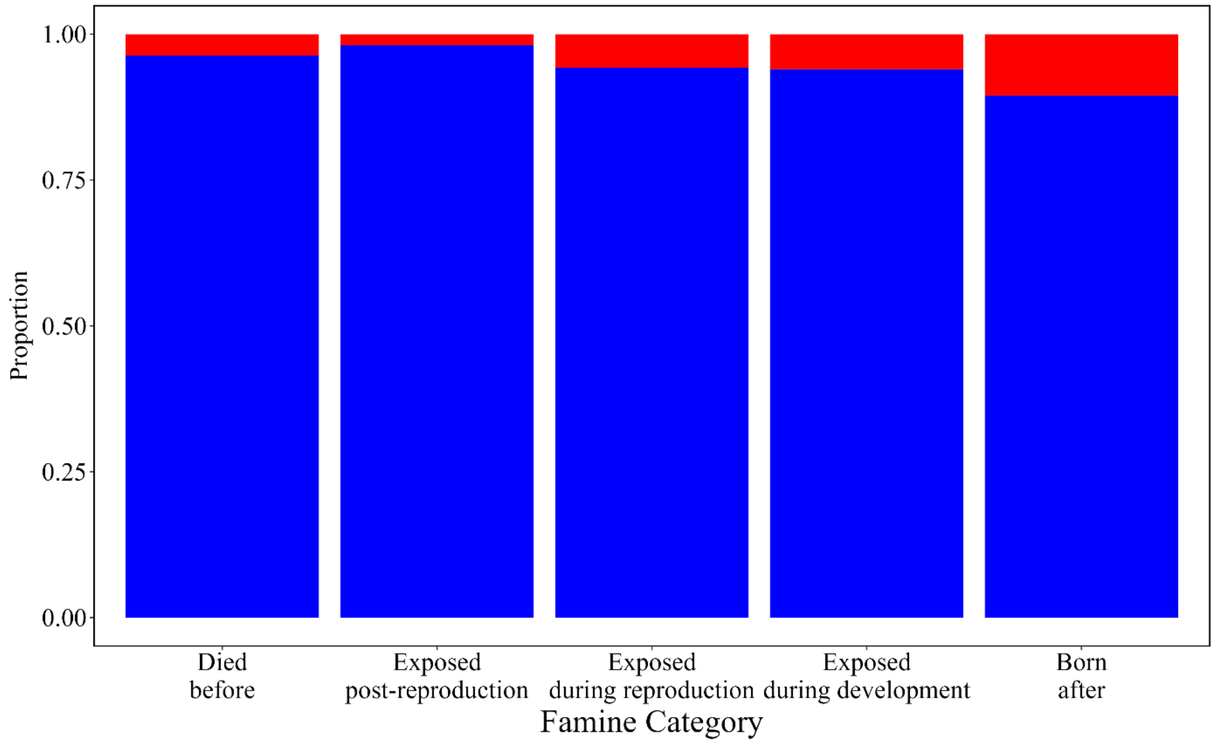

Figure S5: Bar charts showing the proportion of censored (red) vs those with death dates (blue) in each group after censoring biases were removed.

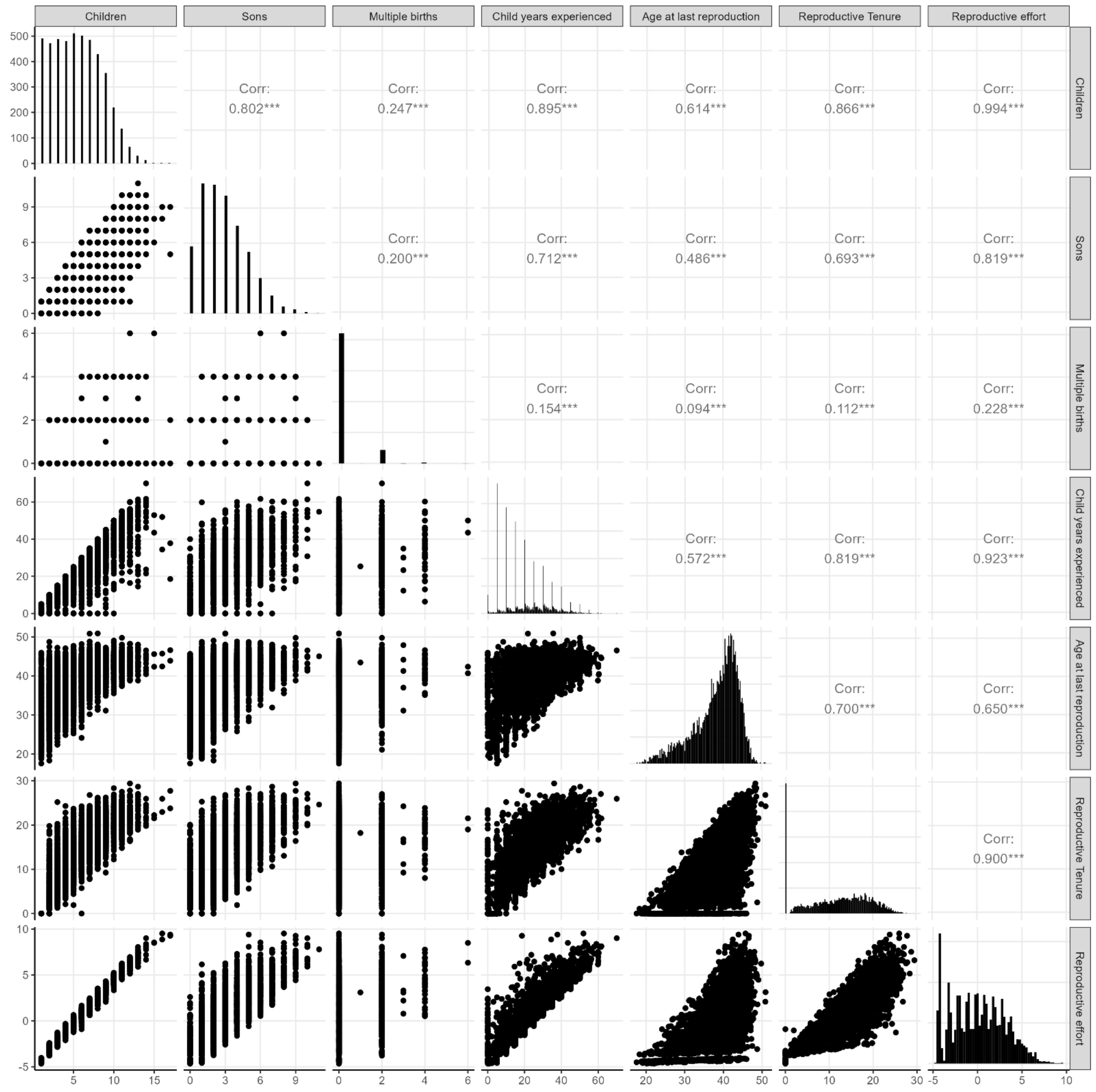

Figure S6: Correlation matrix of reproductive indicators and reproductive effort using *ggally 2.1.2*. The plot shows pairwise relationships between variables: number of children born, number of sons born, number of multiple births, child-years experienced, age at last reproduction, reproductive tenure, and reproductive effort. Pearson correlation coefficients with significance levels shown above diagonal (*p<0.05, **p<0.01, ***p<0.001). Scatterplots showing bivariate relationships below diagonal. Histograms show the distribution of each variable on diagonal.

Table S1: Cox proportional hazards model used for illustrating associations between reproductive effort and lifespan across famine exposure groups shown in Fig. 2. Hazard ratios (β) with 95% confidence intervals (CI), and Wald test p-values are shown for reproductive effort, socioeconomic status (upper and lower compared to intermediate), and relative birth date. Models were fit using *Coxph()* from *survival 3.5.7* without random effects. Significant effects ($p < 0.05$) are in bold.

	Died before famine			Exposed during post-reproduction			Exposed during reproduction			Exposed during development			Born after famine		
Predictors	*β*	*CI*	*p*	*β*	*CI*	*p*	*β*	*CI*	*p*	*β*	*CI*	*p*	*β*	*CI*	*p*
Reproductive effort	1.00	0.98 – 1.02	0.821	1.00	0.97 – 1.02	0.793	**1.04**	**1.02 – 1.07**	**0.001**	1.01	0.99 – 1.04	0.334	1.00	0.98 – 1.02	0.967
Socioeconomic status (upper)	*1.11*	*0.98 – 1.27*	*0.098*	1.08	0.92 – 1.27	0.363	0.97	0.84 – 1.13	0.729	0.96	0.81 – 1.14	0.641	**1.22**	**1.04 – 1.43**	**0.013**
Socioeconomic status (lower)	**1.32**	**1.10 – 1.58**	**0.003**	**1.47**	**1.17 – 1.85**	**0.001**	**1.45**	**1.22 – 1.71**	**<0.001**	0.98	0.79 – 1.20	0.820	1.12	0.93 – 1.34	0.227
Relative birth date	**1.03**	**1.02 – 1.03**	**<0.001**	**1.04**	**1.03 – 1.05**	**<0.001**	1.00	0.99 – 1.01	0.532	1.00	0.98 – 1.01	0.683	**0.99**	**0.98 – 0.99**	**<0.001**

Table S2: Cox proportional hazards mixed-effects models examining the effects of reproductive effort on log-hazard mortality risk not split by famine exposure group with mothers dying before the famine as the baseline exposure group. Analogous to the Structural Equation Model presented in Table 1, but accounting for among-family, birth year, and regional variance. Fixed effects show log-hazard risk (β) and standard errors (SE); random effects show estimated variance. Chi-square values and p-values were calculated using likelihood ratio tests comparing models with and without the effect and were done only for random effects and interactions of fixed effects with famine exposure groups.

	Died before famine		
Predictors	β (SE)	Chi-sq	p
Fixed effects			
Lifetime reproductive effort	1.003 (0.012)		
Famine cohort (post-reproductive)	0.2 (0.138)		
Famine cohort (reproductively active)	0.577 (0.108)		
Famine cohort (developing)	0.49 (0.121)		
Famine cohort (born after)	0.34 (0.106)		
Relative birth date	1.028 (0.002)		
Socioeconomic status group (upper)	1.019 (0.08)		
Socioeconomic status group (lower)	1.244 (0.113)		
**Lifetime reproductive effort x Famine cohort (post-reproductive)**	**0.99 (0.02)**	**10.844**	**0.028**
**Lifetime reproductive effort x Famine cohort (reproductively active)**	**1.045 (0.018)**		
**Lifetime reproductive effort x Famine cohort (developing)**	**1.012 (0.02)**		

**Lifetime reproductive effort x Famine cohort (born after)**	**0.987 (0.019)**		
**Relative birth date x Famine cohort (post-reproductive)**	**1.01 (0.008)**	**89.285**	**<0.001**
**Relative birth date x Famine cohort (reproductively active)**	**0.97 (0.008)**		
**Relative birth date x Famine cohort (developing)**	**0.97 (0.012)**		
**Relative birth date x Famine cohort (born after)**	**0.953 (0.005)**		
**Socioeconomic status group (upper) x Famine cohort (post-reproductive)**	**0.994 (0.125)**	**33.274**	**0.001**
**Socioeconomic status group (upper) x Famine cohort (reproductively active)**	**0.87 (0.117)**		
**Socioeconomic status group (upper) x Famine cohort (developing)**	**0.806 (0.132)**		
**Socioeconomic status group (upper) x Famine cohort (born after)**	**1.303 (0.125)**		
**Socioeconomic status group (lower) x Famine cohort (post-reproductive)**	**1.01 (0.175)**		
**Socioeconomic status group (lower) x Famine cohort (reproductively active)**	**1.177 (0.151)**		
**Socioeconomic status group (lower) x Famine cohort (developing)**	**0.743 (0.167)**		
**Socioeconomic status group (lower) x Famine cohort (born after)**	**1.003 (0.157)**		
Random effects			
**Family**	**0.185**	**20.994**	**<0.001**

**Birth year**	**0.061**	**36.865**	**<0.001**
**Region**	**0.071**	**77.251**	**<0.001**

Table S3: Cox proportional hazards mixed-effects models examining the effects of reproductive effort on log-hazard mortality risk, stratified by famine exposure group. Five separate models are presented: (1) died before famine, (2) exposed post-reproduction, (3) exposed during reproduction, (4) exposed during development, and (5) born after famine. All models account for among-family, birth year, and regional variance. Fixed effects show log-hazard risk (*β*) and standard errors (SE); random effects show estimated variance. Chi-square values and p-values were calculated using likelihood ratio tests comparing models with and without the effect. For socioeconomic status, Chi-sq tests show the combined effect.

	Died before famine			Exposed during post-reproduction			Exposed during reproduction			Exposed during development			Born after famine		
Predictors	*β (SE)*	*Chi-sq*	*p*	*β (SE)*	*Chi-sq*	*p*	*β (SE)*	*Chi-sq*	*p*	*β (SE)*	*Chi-sq*	*p*	*β (SE)*	*Chi-sq*	*p*
Fixed effects															
Reproductive effort	1.007 (0.011)	0.373	0.542	0.989 (0.017)	0.415	0.519	**1.029 (0.013)**	**4.52**	**0.033**	1.006 (0.016)	0.165	0.685	0.992 (0.015)	0.32	0.571
Relative birth date	**1.033 (0.003)**	**75.604**	**<0.001**	**1.051 (0.006)**	**42.413**	**<0.001**	1.010 (0.006)	2.714	0.099	0.992 (0.007)	1.05	0.306	**0.988 (0.005)**	**6.746**	**0.009**
Socioeconomic status (upper)	*1.061 (0.074)*	*5.284*	*0.071*	*1.032 (0.104)*	*5.429*	*0.066*	0.922 (0.086)	11.532	0.003	0.897 (0.108)	1.17	0.557	1.172 (0.100)	2.725	0.256
Socioeconomic status (lower)	1.279 (0.106)	NA	NA	1.402 (0.147)	NA	NA	1.293 (0.099)	NA	NA	0.984 (0.120)	NA	NA	1.134 (0.110)	NA	NA

Random effects															
Family	<0.001	0.823	0.364	**0.309**	**5.331**	**0.021**	0.15	3.113	0.078	0.157	1.657	0.198	**0.206**	**5.419**	**0.02**
Birth year	**0.41**	**122.668**	**<0.001**	0.005	0.056	0.813	0.011	1.051	0.305	<0.001	0.005	0.945	**0.038**	**4.369**	**0.037**
Region	**0.02**	**4.909**	**0.027**	**0.122**	**15.733**	**<0.001**	**0.187**	**98.946**	**<0.001**	**0.571**	**26.181**	**<0.001**	**0.288**	**55.979**	**<0.001**

Table S4: Pairwise comparisons of the association between lifetime reproductive effort and mortality risk across famine exposure groups, based on Cox proportional hazards mixed-effects models. Diagonal values (bold) show the log-hazard risk (SE) for the effect of reproductive effort on mortality within each group. Off-diagonal values show p-values for pairwise differences in this association between groups. P-values were calculated using likelihood ratio tests, comparing models with two exposure groups in turn, with and without an interaction between reproductive effort and the two exposure groups. Significant differences ($p<0.05$) are highlighted in bold. NA indicates comparisons not applicable due to the structure of the analysis.

	Died before famine	Exposed post-reproduction	Exposed during reproduction	Exposed during development	Born after famine
Died before famine	1.007 (0.011)	NA	NA	NA	NA
Exposed post-reproduction	0.710	0.989 (0.017)	NA	NA	NA
Exposed during reproduction	**0.017**	**0.003**	**1.029 (0.013)**	NA	NA
Exposed during development	0.547	0.263	0.122	1.006 (0.016)	NA
Born after famine	0.735	0.661	**0.007**	0.273	0.992 (0.015)

Table S5: Full Structural equation model with non-significant socioeconomic status groups interactions with reproductive effort on lifespan (as measured by log-hazard mortality risk) from 4,684 mothers. Factor loadings, mean values (Intercept), and residual variance for indicator variables used to estimate lifetime reproductive effort in the measurement model. Estimates are shown with 95% confidence intervals and p-values. Significant effects ($p < 0.05$) are in bold. Effects of famine exposure are compared to women who died before the famine. Socioeconomic statuses are compared to intermediate statuses. Relative birth date is scaled by year and centered by the middle birth date for each group.

Variable	Estimate (95% CI)	P-value
Lifetime reproductive effort loadings		
Number of children born	1 (1-1)	-
**Number of sons born**	**0.536 (0.523-0.548)**	**<0.001**
**Age at last reproduction**	**1.238 (1.187-1.289)**	**<0.001**
**Reproductive tenure**	**2.131 (2.092-2.17)**	**<0.001**
**Child-years experienced**	**3.878 (3.811-3.945)**	**<0.001**
**Number of multibirth children born**	**0.052 (0.044-0.059)**	**<0.001**
Effects on reproductive effort		
Exposure group (exposed post-reproduction)	0.125 (-0.441-0.692)	0.664
Exposure group (exposed during reproduction)	*0.387 (-0.006-0.779)*	*0.053*
**Exposure group (exposed during development)**	**0.552 (0.081-1.022)**	**0.022**
**Exposure group (born after)**	**-0.503 (-0.921--0.085)**	**0.018**
Relative birth date	-0.004 (-0.013-0.004)	0.343
Relative birth date x Exposure group (exposed post-reproduction)	0.022 (-0.005-0.049)	0.108
**Relative birth date x Exposure group (exposed during reproduction)**	**0.033 (0.008-0.057)**	**0.01**

Relative birth date x Exposure group (exposed during development)	0.019 (-0.018-0.057)	0.307
**Relative birth date x Exposure group (born after)**	**-0.061 (-0.078--0.044)**	**<0.001**
**Socioeconomic status (lower)**	**-0.54 (-1.019--0.061)**	**0.027**
Socioeconomic status (lower) x Exposure group (exposed post-reproduction)	-0.471 (-1.228-0.287)	0.223
**Socioeconomic status (lower) x Exposure group (exposed during reproduction)**	**-0.654 (-1.3--0.009)**	**0.047**
Socioeconomic status (lower) x Exposure group (exposed during development)	-0.133 (-0.875-0.61)	0.726
Socioeconomic status (lower) x Exposure group (born after)	-0.336 (-1.011-0.339)	0.329
**Socioeconomic status (upper)**	**0.61 (0.244-0.976)**	**0.001**
Socioeconomic status (upper) x Exposure group (exposed post-reproduction)	-0.01 (-0.594-0.573)	0.972
Socioeconomic status (upper) x Exposure group (exposed during reproduction)	-0.114 (-0.656-0.428)	0.680
Socioeconomic status (upper) x Exposure group (exposed during development)	-0.266 (-0.891-0.359)	0.404
**Socioeconomic status (upper) x Exposure group (born after)**	**-0.583 (-1.158--0.009)**	**0.047**
Effects on mortality risk		
**Exposure group (exposed post-reproduction)**	**0.334 (0.283-0.393)**	**<0.001**
**Exposure group (exposed during reproduction)**	**0.694 (0.593-0.812)**	**<0.001**
**Exposure group (exposed during development)**	**0.6 (0.507-0.711)**	**<0.001**
**Exposure group (born after)**	**0.462 (0.396-0.538)**	**<0.001**
Lifetime reproductive effort	0.998 (0.973-1.024)	0.906

Lifetime reproductive effort x Socioeconomic status group (higher)	0.979 (0.95-1.009)	0.165
Lifetime reproductive effort x Socioeconomic status group (lower)	1.011 (0.987-1.036)	0.370
Lifetime reproductive effort x Exposure group (exposed post-reproduction)	0.996 (0.968-1.025)	0.780
**Lifetime reproductive effort x Exposure group (exposed during reproduction)**	**1.045 (1.013-1.077)**	**0.005**
Lifetime reproductive effort x Exposure group (exposed during development)	1.013 (0.981-1.046)	0.439
Lifetime reproductive effort x Exposure group (born after)	1.001 (0.969-1.035)	0.960
**Relative birth date**	**1.026 (1.022-1.029)**	**<0.001**
Relative birth date x Exposure group (exposed post-reproduction)	1.005 (0.998-1.012)	0.166
**Relative birth date x Exposure group (exposed during reproduction)**	**0.97 (0.962-0.98)**	**<0.001**
**Relative birth date x Exposure group (exposed during development)**	**0.972 (0.961-0.984)**	**<0.001**
**Relative birth date x Exposure group (born after)**	**0.96 (0.954-0.967)**	**<0.001**
**Socioeconomic status (lower)**	**1.287 (1.08-1.534)**	**0.005**
Socioeconomic status (lower) x Exposure group (exposed post-reproduction)	1.027 (0.811-1.303)	0.82
Socioeconomic status (lower) x Exposure group (exposed during reproduction)	1.123 (0.872-1.448)	0.369
**Socioeconomic status (lower) x Exposure group (exposed during development)**	**0.763 (0.587-0.991)**	**0.043**
Socioeconomic status (lower) x Exposure group (born after)	0.862 (0.67-1.108)	0.246

Socioeconomic status (upper)	1.105 (0.969-1.259)	0.133
Socioeconomic status (upper) x Exposure group (exposed post-reproduction)	0.962 (0.798-1.161)	0.685
Socioeconomic status (upper) x Exposure group (exposed during reproduction)	0.868 (0.71-1.061)	0.165
Socioeconomic status (upper) x Exposure group (exposed during development)	0.868 (0.702-1.073)	0.191
Socioeconomic status (upper) x Exposure group (born after)	1.13 (0.918-1.391)	0.251
Intercepts		
**Number of children born**	**5.264 (4.977-5.551)**	**<0.001**
**Number of sons born**	**2.695 (2.539-2.851)**	**<0.001**
**Age at last reproduction**	**38.132 (37.761-38.503)**	**<0.001**
**Reproductive tenure**	**11.87 (11.252-12.488)**	**<0.001**
**Child-years experienced**	**21.127 (20.012-22.242)**	**<0.001**
**Number of multibirth children born**	**0.211 (0.187-0.235)**	**<0.001**
Residual variances		
**Number of children born**	**0.343 (0.276-0.411)**	**<0.001**
**Number of sons born**	**1.348 (1.283-1.412)**	**<0.001**
**Age-at-last reproduction**	**19.315 (18.463-20.166)**	**<0.001**
**Reproductive tenure**	**10.772 (10.128-11.415)**	**<0.001**
**Child-years experienced**	**27.371 (24.86-29.881)**	**<0.001**
**Number of multibirth children born**	**0.439 (0.397-0.481)**	**<0.001**
**Lifetime reproductive effort**	**8.298 (7.995-8.602)**	**<0.001**

Table S6: Descriptive statistics of key variables for mothers analyzed in the structural equation model, stratified by famine exposure group. Values are presented as mean (standard deviation) and range. Groups were: died before famine (n = 1,297), exposed post-reproduction (n = 705), exposed during reproduction (n = 1,044), exposed during development (n = 716), born after famine (n = 922), and global sample (n = 4,684). Variables include lifespan, censoring status, reproductive measures (number of children, sons, multiple births), child-years experienced, age at last reproduction, reproductive tenure, socioeconomic status, birthdate, and date of last appearance, which includes dates of last seen or death dates.

	Died before (n = 1297)	Exposed post-reproduction (n = 705)	Exposed during reproduction (n = 1044)	Exposed during development (n = 716)	Born after (n = 922)	Global (n = 4684)
**Lifespan (including censored)**						
Mean (SD)	63.654 (10.864)	73.406 (9.403)	66.883 (12.235)	69.687 (12.518)	69.851 (12.772)	67.983 (12.096)
Range	44.899 - 93.431	47.986 - 99.448	44.992 - 99.559	44.937 - 98.458	45.079 - 103.227	44.899 - 103.227
**Censoring status (censored, 0, death date, 1)**						
Mean (SD)	0.964 (0.187)	0.976 (0.154)	0.943 (0.233)	0.940 (0.238)	0.895 (0.307)	0.944 (0.231)
Range	0.000 - 1.000	0.000 - 1.000	0.000 - 1.000	0.000 - 1.000	0.000 - 1.000	0.000 - 1.000
**Children born**						
Mean (SD)	5.551 (3.156)	5.756 (2.891)	5.547 (2.934)	5.758 (2.994)	4.764 (2.963)	5.458 (3.025)
Range	1.000 - 17.000	1.000 - 14.000	1.000 - 14.000	1.000 - 14.000	1.000 - 14.000	1.000 - 17.000
**Sons born**						
Mean (SD)	2.833 (1.990)	2.960 (1.994)	2.810 (1.950)	2.915 (1.954)	2.523 (1.929)	2.798 (1.969)
Range	0.000 - 10.000	0.000 - 11.000	0.000 - 10.000	0.000 - 10.000	0.000 - 10.000	0.000 - 11.000

**Multiple births**						
Mean (SD)	0.335 (0.832)	0.177 (0.602)	0.187 (0.617)	0.156 (0.578)	0.181 (0.621)	0.221 (0.680)
Range	0.000 - 6.000	0.000 - 4.000	0.000 - 4.000	0.000 - 4.000	0.000 - 4.000	0.000 - 6.000
**Child years experienced**						
Mean (SD)	21.341 (11.875)	22.695 (12.084)	22.036 (12.263)	24.185 (13.136)	20.033 (13.783)	21.877 (12.643)
Range	0.000 - 57.866	0.000 - 55.044	0.000 - 61.660	0.000 - 61.384	0.000 - 70.000	0.000 - 70.000
**Age at last reproduction**						
Mean (SD)	38.797 (5.368)	39.608 (5.164)	38.799 (5.524)	38.537 (5.634)	36.214 (6.356)	38.371 (5.728)
Range	19.105 - 49.763	19.872 - 49.043	17.553 - 50.838	18.759 - 48.022	18.308 - 50.888	17.553 - 50.888
**Reproductive Tenure**						
Mean (SD)	12.289 (7.197)	13.088 (6.843)	12.715 (7.020)	13.078 (6.951)	10.549 (7.210)	12.282 (7.126)
Range	0.000 - 27.748	0.000 - 26.562	0.000 - 29.410	0.000 - 28.686	0.000 - 28.351	0.000 - 29.410
**Socioeconomic status**						
Mean (SD)	1.583 (0.722)	1.706 (0.719)	1.843 (0.772)	1.818 (0.788)	1.870 (0.773)	1.752 (0.762)
Range	1.000 - 3.000	1.000 - 3.000	1.000 - 3.000	1.000 - 3.000	1.000 - 3.000	1.000 - 3.000
**Birthdate**						
Mean (SD)	1776.960 (20.898)	1811.581 (8.173)	1834.726 (7.496)	1858.337 (6.147)	1886.975 (12.280)	1829.141 (42.133)
Range	1725.301 - 1819.164	1783.159 - 1822.356	1822.392 - 1848.077	1848.126 - 1869.381	1869.414 - 1910.995	1725.301 - 1910.995

Date of last appearance

Mean (SD)	1840.613 (19.523)	1884.987 (9.659)	1901.608 (14.053)	1928.024 (14.364)	1956.826 (18.435)	1897.124 (44.952)
Range	1779.151 - 1865.063	1869.490 - 1914.000	1870.000 - 1943.641	1894.000 - 1961.033	1915.367 - 2011.816	1779.151 - 2011.816
